# Implementation of clinical practice guidelines on lifestyle interventions in Swedish primary healthcare – a two-year follow up

**DOI:** 10.1186/s12913-018-3023-z

**Published:** 2018-04-02

**Authors:** Therese Kardakis, Lars Jerdén, Monica E. Nyström, Lars Weinehall, Helene Johansson

**Affiliations:** 1 Department of Public Health and Clinical Medicine, Epidemiology and Global Health, Umeå University, 90185 Umeå, SE Sweden; 2 Department of Learning, Informatics, Management and Ethics, Medical Management Centre, Karolinska Institutet, 17177 Stockholm, SE Sweden; 3 Center for Clinical Research Dalarna, Nissers väg 3, 79172 Falun, SE Sweden; 4 School of Education, Health and Social Studies, Dalarna University, 79188 Falun, SE Sweden

**Keywords:** Implementation, Lifestyle, Clinical practice guidelines, Primary health care, Preventive health services, Health promotion, Smoking, Counselling

## Abstract

**Background:**

Implementation of interventions concerning prevention and health promotion in health care has faced particular challenges resulting in a low frequency and quality of these services. In November 2011, the Swedish National Board of Health and Welfare released national clinical practice guidelines to counteract patients’ unhealthy lifestyle habits. Drawing on the results of a previous study as a point of departure, the aim of this two-year follow up was to assess the progress of work with lifestyle interventions in primary healthcare as well as the uptake and usage of the new guidelines on lifestyle interventions in clinical practice.

**Methods:**

Longitudinal study among health professionals with survey at baseline and 2 years later. Development over time and differences between professional groups were calculated with Pearson chi-square test.

**Results:**

Eighteen percent of the physicians reported to use the clinical practice guidelines, compared to 58% of the nurses. Nurses were also more likely to consider them as a support in their work than physicians did. Over time, health professionals usage of methods to change patients’ tobacco habits and hazardous use of alcohol had increased, and the nurses worked to a higher extent than before with all four lifestyles. Knowledge on methods for lifestyle change was generally high; however, there was room for improvement concerning methods on alcohol, unhealthy eating and counselling. Forty-one percent reported to possess thorough knowledge of counselling skills.

**Conclusions:**

Even if the uptake and usage of the CPGs on lifestyle interventions so far is low, the participants reported more frequent counselling on patients’ lifestyle changes concerning use of tobacco and hazardous use of alcohol. However, these findings should be evaluated acknowledging the possibility of selection bias in favour of health promotion and lifestyle guidance, and the loss of one study site in the follow up. Furthermore, this study indicates important differences in physicians and nurses’ attitudes to and use of the guidelines, where the nurses reported working to a higher extent with all four lifestyles compared to the first study. These findings suggest further investigations on the implementation process in clinical practice, and the physicians’ uptake and use of the CPGs.

**Electronic supplementary material:**

The online version of this article (10.1186/s12913-018-3023-z) contains supplementary material, which is available to authorized users.

## Background

Introducing new clinical practice guidelines (CPGs) and implementing changes in healthcare practices is described as complex and challenging, and often the intended result is not achieved [1]. A range of known factors influences the implementation of guidelines in healthcare. These factors consider the field and characteristics of the innovation; the adopters; the context or setting; and the specific implementation activities [2–4]. It is important to be aware and recognise these when planning for implementation since they can both obstruct and facilitate the uptake of new practices.

The introduction of interventions in the field of health orientation in health care, i.e. prevention and health promotion, has faced specific challenges resulting in a low frequency and quality of these services [5–8]. Health-oriented healthcare can be defined as: “a perspective that focuses on health promotion and prevention, which can be applied comprehensively in a healthcare system and which should be a baseline for all patient care and treatment” [9]. One of the specific challenges is that prevention and health promotion interventions are often not considered to have priority and healthcare professionals are ambiguous about how to prioritise [10, 11]. A study of general practitioners showed that their delivery of care was dominated by diagnosing and treating disease [12]. Other studies explain this as traditional medical care being prioritised “on the cost of health promotion” [13, 14]. Moreover, an important character of health orientation refers to the counselling situation and ethics. Health care professionals can consider lifestyle counselling as problematic since it concerns how people choose to live their lives and it can be perceived as paternalistic [15]. Furthermore, research on health physicians’ attitudes to health orientation shows differing results. In one study the physicians were found positive to health promotion and prevention [16]; in another they were found positive to prevention and negative to health promotion [10], and in still another the physicians represented a range of attitudes – from ignoring the field to being a nurturer of the same [6]. Nurses, on the other hand, show similar results in various studies and have been found to be positive to the health-oriented perspective [6, 10, 17]. Physicians´ relatively lower interest in health orientation has been explained as being a part of their professional identity and in accordance to current norms, i.e. to value rare medical fields higher than every day common health issues [12, 18]. Studies have also shown structural obstacles to health orientation such as lack of time [10, 15, 19, 20], ambiguous protocols, and lack of appropriate structures and referral options [11, 19, 20]. Moreover, knowledge gaps have been identified as hindering the implementation [11, 15, 20].

In line with governmental efforts to promote health orientation, the Swedish National Board of Health and Welfare (NBHW) published national CPGs in November 2011 [21]. The guidelines include methods to counteract tobacco use, hazardous use of alcohol, unhealthy eating habits and insufficient physical activity, and were to be implemented in Sweden’s 21 regional health care organisations [21]. In short, the recommended methods were divided into three different levels – brief advice, counselling and advanced counselling. All levels presume that healthcare professionals possess sufficient knowledge about necessary healthy lifestyle habits and counselling methods. It is also presumed that a persons’ unhealthy lifestyle has been successfully identified, which means healthcare professionals also need to know when and how to ask questions concerning patient lifestyles [21]. The official name of the guidelines is: “National guidelines for methods of preventing disease”, hereafter referred to as “CPGs on lifestyle interventions”. In a previous study, we investigated the extent of health care professionals’ knowledge-, attitudes-, perceived organizational support-, and work with *lifestyle interventions* prior to the release of the guidelines [22]. The main finding was that health professionals (physicians and nurses) to a large extent had a positive attitude to and thorough overall knowledge about methods to aid changes of patients’ lifestyles. However, both knowledge of different lifestyle intervention methods and the extent of involvement in the promotion of patients’ lifestyles differed between professional groups and according to years of professional experience. Management was perceived as supportive to primary healthcare staff’s work with patients’ lifestyle, but scarce collaboration with other stakeholders was detected.

The purpose of the present paper was to investigate the situation 2 years later. The aim was to assess the progress of the work with lifestyle interventions in primary healthcare, as well as the uptake and usage of the CPGs on lifestyle interventions in clinical practice.

## Methods

A longitudinal study with questionnaires distributed to physicians and nurses twice over a period of 2 years.

### Setting

The Swedish healthcare system is organised at national, regional and local levels. At the national level, the Ministry of Health and Social Affairs establishes principles and guidelines with the help of governmental agencies, e.g. NBHW. At the regional level, the financing and provision of healthcare to all citizens is the responsibility of 21 healthcare organisations, run by directly elected county councils. At the local level, municipalities are responsible for home-based healthcare. Healthcare is primarily funded by taxes, supplemented by governmental grants and user fees [23]. Private provision of healthcare is getting more common in primary healthcare, still financed by the county councils [24].

### Sample and surveys

In the first study [22], two healthcare organisations were purposively selected for accessibility and comparability representing sparsely populated regions in the north of Sweden. One of the organisations decided to withdraw their participation in the follow-up study due to on-going organisational changes.

Data collection consisted of two questionnaires with close-ended questions. The statements in the web-based questionnaire was informed by models of implementation theory guided by Damschroder [2] and Greenhalgh [3]. Specific statements regarding health orientation were influenced by a theory developed by Johnson and Paton that focuses on change management for reorienting health services [25]. We pilot tested the Questionnaire I on a sample of eight persons to assess its face validity. The piloting resulted in a reduced number of statements and in clarifying some of the vocabulary. The structured web-questionnaires were distributed as a link in an “invitation-to-participate” e-mail directly to *all* registered nurses and physicians employed in primary healthcare (PHC) within the organisations (*n* = 645). Questionnaire I (Additional file 1) was distributed in May 2011 (before the release of the guidelines) and Questionnaire II in May 2013 (18 months after the release). Questionnaire I received a response rate of 49% [22]. The second questionnaire was distributed to the respondents of questionnaire I in the remaining participating healthcare organisation (*n* = 223), and a response rate of 50% was obtained (72 nurses and 39 physicians). Two years between the studies were considered a reasonable amount of time for a follow up on the initial implementation of the CPGs. A five-point Likert scale was used for the close-ended responses (item responses ranged from “completely disagree”, “partly disagree”, “partly agree”, “agree to a high extent” to “completely agree”). In the second questionnaire, the objective of seven new questions was to investigate the uptake in clinical practice of the CPGs. A part from this change, Questionnaire II contained the same statements as Questionnaire I with some smaller modifications. An open-ended exploratory question focusing on perceived implementation difficulties was also included: “*At my Primary health care centre (PHCC) the following factors/processes/support etc. presents an obstacle to the implementation of the national guidelines on disease prevention methods*”.

### Data analyses

The response alternatives “completely agree” and “agree to a high extent” were clustered into one category, and considered as a positive response to the statements. Differences between professional groups, years in profession, and gender in the physician group (the group of nurses consisted almost only by women) were analysed, both for the respective questionnaires, and for the difference between the first and the second questionnaire. Statistical significance was examined with Pearson chi-square test, *p*-values < 0.05 were considered as significant. No statistical significant gender differences were found and gender analyses are not presented in the results. Qualitative data from the open question regarding difficulties were analysed with an inductive approach by using thematic content analyses [26]. The answers were read through several times to make sense of the text to identify relevant units for analysis, that is, responses that captured perceived difficulties to the implementation of the CPGs. The units were summarized into themes of difficulties and described to exemplify the quantitative data [26]. The first author performed the analyses, and other two researchers (LJ and HJ) read the answers and checked the analyses independently. Finally, the data were translated from Swedish to English by the first author (TK).

## Results

**Fig. 1:**
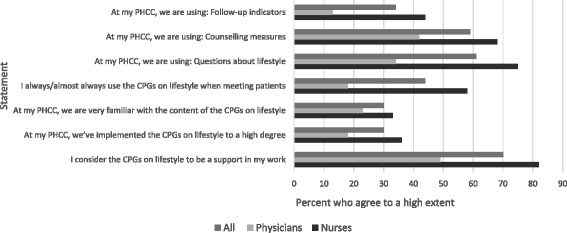
Progress of the implementation of CPGs on lifestyle interventions in PHC

The first part of the result (Fig. 1) focuses on the uptake and usage of the specific intervention i.e. the CPGs on lifestyle interventions. The second part of the result (Tables 1, 2, 3, 4) describes the two-year follow up of the development of working with lifestyle interventions in Swedish primary healthcare.

**Table 1:** Attitudes toward promotion of healthy lifestyles, percent of health care professionals who agree to a high extent

Statements	At my PHCC, we consider it important to promote healthy lifestyles for our patients					At my PHCC, we work extensively with the promotion of healthy lifestyles among our patients				
Questionnaire	Q I		Q II		Q I → Q II	Q I		Q II		Q I → Q II
Characteristics	Percent	P	Percent	P	P	Percent	P	Percent	P	P
Profession		0.774		0.961			0.447		0.051	
Nurses	81		88		0.232	53		65		0.094
Physicians	80		87		0.309	48		46		0.856
Years in profession		0.987		0.215			0.042		0.044	
0–10	81		81		0.926	43		44		0.918
> 10	81		90		0.072	57		65		0.264
Total	81		87		0.119	52		59		0.229
Statements	I perceive tasks like promotion of healthy lifestyles as compatible with overall PHC aims and objectives					According to me, there is a need to develop work with health promotion in PHC				
Questiuonnaire	Q I		Q II		Q I → Q II	Q I		Q I		Q I → Q II
Characteristics	Percent	P	Percent	P	P	Percent	P	Percent	P	P
Profession		0.915		0.314			0.061		0.002	
Nurses	87		89		0.714	90		92		0.670
Physicians	88		82		0.418	81		69		0.166
Years in profession		0.798		0.101			0.921		0.030	
0–10	87		78		0.266	87		72		0.166
> 10	88		90		0.639	87		89		0.064
Total	87		87		0.829	87		84		0.446

*PHCC* Primary health care centre

*PHC* Primary health care

*QI* Questionnaire I (May, 2011)

*QII* Questionnaire II (May, 2013)

### Uptake and usage of CPGs on lifestyle interventions

The implementation of the CPGs was assessed by seven statements (Fig. 1) and by one open-ended question, which aimed to capture the perceived difficulties of the implementation.

Less than a third of the health professionals reported that the CPGs had been implemented at the PHCC to a high degree, and that they were very familiar with the content (Fig. 1). Seventy percent of the respondents considered the CPGs as a support in their work, and 44 % reported to always use the CPGs when meeting patients. Differences were seen across the health professional groups, where only 18% of the physicians reported to use the CPGs compared to 58% of the nurses (*P* = < 0.001). Nurses were also more likely to consider them as a support in their work than physicians did (*P* = < 0.001).

The CPGs include three kinds of support tools, and the health professionals reported the use of them at the PHCC as follows: questions about lifestyles (61%), counselling measures (59%), and follow-up indicators (34%). Discrepancies could be seen between nurses and physicians in the usage of all three support-tools, where physicians constantly reported a lower use: questions about lifestyles (*P* = < 0.001), counselling measures (*P* = 0.009), and follow-up indicators (*P* = 0.004).

### Perceived difficulties to implement the CPGs

The analyses of the open-ended question resulted in four themes focusing on the lack of: 1) Resources 2) Organisational support 3) Priority and 4) Unfavourable organisational climate. Both physicians and nurses emphasized the theme about resources concerning lack of time and heavy workload.

#### Lack of resources

The health professionals referred to a general lack of resources. One respondent said: “*The fact that we haven’t received any extra resources complicates it…we have to do this on top of all the rest”.* Both physicians and nurses stated that the time available to reflect over routines, study the guidelines and work with the implementation in practice, was limited. Furthermore, the counselling measures were perceived as too time consuming in the daily routine. As one respondent put it: “*It is difficult to find the time to carry out the counselling measures…a prescription on anti-hypertensions is faster than a conversation on lifestyle”* and another: *“…shortage of resources to listen to patients’ own thoughts concerning improvement of lifestyle, and a normal appointment is too short”.* Workload was reported to be very heavy, with many new tasks in primary care, as well as a high number of patients and lots of administration. The workload was partly due to the shortage of health professionals, especially physicians, which created a “spill-over effect” on other health professionals. One respondent expressed: “*Many new tasks have been added primary care, but no increase in the number of health professionals”,* and *“We have a shortage of physicians resulting in a high workload on the available ones, with a spill-over effect on us all”.* The lack of resources, and to keep up with a heavy workload with a consistent shortage of physicians, were reported to cause a feeling of lack of energy and strength to work with development and improvements. As one respondent put it: “*The workload is very high and there is no time to work with new routines”*.

#### Lack of organisational support

The respondents stated lack of support from management concerning the CPG implementation, in particular by the nurses. It was perceived as nobody took the responsibility for the implementation, and that the information about the guidelines had been poor. Management was also considered as not being interested, not available, and unexperienced. As one respondent described: *“It feels like nobody takes the responsibility for the implementation, we have received information about the CPGs at our PHC meetings and we know about their existence, but…”,* and: *“No manager is available who think this is important”.* Limited education on the guidelines, and difficulties to organize continuous education alternatives for different health professionals was also reported about as an obstacle.

Moreover, some features in the organisation’s structure and functioning were perceived to influence implementation, such as the size of PHCC’s (large with many employees), the medical record not being adapted to the CPGs, and the loss of continuity given the constant use of temporary physicians. The following comments illustrate these perceptions: “*an imperfect and time-consuming digital system*”, and another*: “temporary physicians are not updated, or willing to work with this”.*

#### Lack of priority

The lack of priority of the CPGs on lifestyle interventions concerned both the *form* i.e. CPGs and the *content* i.e. lifestyle interventions, as a part of prevention and health promotion within healthcare. Physicians and nurses underlined that focus was to carry out the normal and basic medical services that concentrate on care of already ill patients, and that the prevention field was not being prioritised. For example, one respondent reported: “*patients who are not yet ill have low priority*”, and another: “*focus is to manage the basic health care services”,* and still another: *“It is difficult to prioritize when there are already ill patients who need care”.*

Furthermore, the physicians in particular, referred that the guidelines were not prioritised since much of the work and counselling about patient lifestyles was already being done, even if less structured. It was also reported that it was difficult to prioritise *these* particular guidelines since there are so many all the time, which also bring about a tiredness of guidelines. One respondent commented: *“In primary care there are always numerous new guidelines to relate to…”,* and another: *“…what obstacles (implementation) is health professionals’ tiredness of all national guidelines”,* and still another: *“lifestyle counselling with patients is used when it is medically motivated – independently of the content in any guidelines”.*

#### Unfavourable organisational climate

Features of the organisation climate were reported to impact implementation negatively. Health professionals reported about divergences and lack of collaboration between nurses, physicians and managers; an aged physician group with an outdated traditional view of treatments; as well as low commitment to the CPGs by some co-workers. As one respondent put it: “…*divergences between professional groups which should collaborate”.* Furthermore, the respondents referred to a numerous and heterogeneous group of co-workers. It was considered difficult to “have all on board”, i.e. to work according to the guidelines. Some co-workers were not interested, while others did not consider prevention important. Example of comments were: “*There are many coworkers one needs to secure changes and guidelines with”*, and another: “*The group of physicians are generally negative”*, and still another: “A*ll health professionals don’t consider prevention to be important”.*

### Follow-up of work with prevention and health promotion in primary care

Comparing questionnaire I and II some significant developments over time were found. Among other things, the inconsistencies between nurses and physicians have increased. However, for the overall PHC not that much had changed.

#### Attitudes

No statistically significant changes over time were found (Table 1). In the second measurement, the health professionals confirmed their previous statements - to consider it important to work with patients’ lifestyles and that it is compatible with the overall aim of PHC. Fifty-nine percent reported that they as a group worked extensively with patients’ lifestyles, but this differed depending on profession and years in profession. Eighty-four percent considered that there is still a need to develop health promotion work in PHC. Nurses were more likely to report this need than physicians were, as were health professionals with more than 10 years in their profession than those with less experience.

#### Knowledge of methods for lifestyle interventions

There were no significant changes in knowledge from the first measurement (Table 2).

**Table 2:** Knowledge of lifestyle intervention methods for disease prevention, percent of health care professionals who agree to a high extent

Statements	At my PHCC, we have a thorough knowledge of how we can promote healthy lifestyle habits among our patients					I have a thorough knowledge of disease prevention methods concerning tobacco use				
Questionnaire	Q I		Q II		Q I → Q II	Q I		Q II		Q I → Q II
Characteristics	Percent	P	Percent	P	P	Percent	P	Percent	P	P
Profession		0.006		0.007			0.474		0.821	
Nurses	83		86		0.476	71		80		0.133
Physicians	66		64		0.861	75		82		0.417
Years in profession		0.099		0.289			0.051		0.954	
0–10	71		72		0.904	65		81		0.084
> 10	80		81		0.917	77		81		0.498
Total	77		78		0.749	72		81		0.087
Statements	I have a thorough knowledge of disease prevention methods concerning hazardous use of alcohol					I have a thorough knowledge of disease prevention methods concerning unhealthy eating habits				
Questionnaire	Q I		Q II		Q I → Q II	Q I		Q II		Q I → Q II
Characteristics	Percent	P	Percent	P	P	Percent	P	Percent	P	P
Profession		0.951		0.920			0.003		0.000	
Nurses	63		66		0.654	84		93		0.075
Physicians	63		67		0.701	67		67		0.961
Years in profession		0.063		0.163			0.004		0.665	
0–10	55		56		0.895	68		81		0.167
> 10	67		70		0.679	85		85		0.974
Total	63		66		0.554	79		84		0.282
Statements	I have a thorough knowledge of disease prevention methods concerning insufficient physical activity					I have a thorough knowledge of disease prevention methods concerning counselling measures as MI, CBT				
Questionnaire	Q I		Q II		Q I → Q II	Q I		Q II		Q I → Q II
Characteristics	Percent	P	Percent	P	P	Percent	P	Percent	P	P
Profession		0.438		0.234		Not measured			0.176	Not measured
Nurses	82		90		0.146			37		
Physicians	78		82		0.620			50		
Years in profession		0.117		0.485					0.223	
0–10	76		91		0.146			50		
> 10	84		86		0.620			38		
Total	81		87		0.161			41		

*PHCC* Primary health care centre

*QI* Questionnaire I (May, 2011)

*QII* Questionnaire II (May, 2013)

*MI* Motivational interviewing

*CBT* Cognitive behavioral therapy

The respondents, as a group, considered themselves to have a thorough knowledge about how to promote healthy lifestyle habits among their patients. However, nurses believed so to a higher degree than physicians did, which is consistent with the first measurement. Individually, the respondents reported a thorough knowledge for the different lifestyle intervention methods in the following way: insufficient physical activity (87%), unhealthy eating habits (84%), tobacco use (81%) and hazardous use of alcohol (66%). Concerning unhealthy eating habits, nurses reported a statistically significant higher level of knowledge than physicians did, consistent with the findings 2 years earlier. Furthermore, 41% of the respondents reported to have a thorough knowledge in lifestyle counselling (not measured in the first study).

#### Extent of work with lifestyle interventions

There were significant positive changes over time concerning the use of methods for changing patient lifestyle concerning tobacco use and prevention of hazardous use of alcohol (Table 3).

**Table 3:** Extent of work with promotion of healthy lifestyles habits, percent of health care professionals who agree to a high extent

Statements	The promotion of healthy lifestyle habits among my patients is a substantial part of my duties					I work to a great extent with promotion of healthy lifestyle habits concerning tobacco use				
Questionnaire	Q I		Q II		Q I → Q II	Q I		Q II		Q I → Q II
Characteristics	%	P	%	P	P	%	P	%	P	P
Profession		0.007		0.003			0.008		0.704	
Nurses	66		65		0.892	59		79		0.003
Physicians	47		36		0.250	77		76		0.904
Years in profession		0.000		0.276			0.151		0.652	
0–10	44		47		0.774	59		75		0.102
> 10	70		58		0.091	68		79		0.091
Total	60		55		0.389	65		78		0.015
Statements	I work to a great extent with promotion of healthy lifestyle habits concerning hazardous use of alcohol					I work to a great extent with promotion of healthy lifestyle habits concerning unhealthy eating habits				
Questionnaire	Q I		Q II		Q I → Q II	Q I		Q II		Q I → Q II
Characteristics	%	P	%	P	P	%	P	%	P	P
Profession		0.666		0.930			0.592		0.000	
Nurses	50		66		0.033	67		80		0.043
Physicians	53		65		0.252	63		45		0.065
Years in profession		0.088		0.977			0.433		0.236	
0–10	44		66		0.037	62		59		0.781
> 10	56		65		0.176	67		71		0.580
Total	51		65		0.016	66		68		0.701
Statements	I work to a great extent with promotion of healthy lifestyle habits concerning insufficient physical activity					I would like to work to a greater extent with promotion of healthy lifestyles among my patients				
Questionnaire	Q I		Q II		Q I → Q II	Q I		Q II		Q I → Q II
Characteristics	%	P	%	P	P	%	P	%	P	P
Profession		0.099		0.116			0.151		0.001	
Nurses	69		82		0.046	81		83		0.684
Physicians	80		68		0.199	73		54		0.046
Years in profession		0.461		0.741			0.198		0.760	
0–10	70		75		0.562	83		75		0.615
> 10	74		78		0.532	76		72		0.682
Total	72		77		0.364	78		73		0.282

*QI* Questionnaire I (May, 2011)

*QII* Questionnaire II (May, 2013)

Nurses reported to work to a significant higher extent with all four lifestyles compared to the previous study. The extent to which the health professionals reported to work with unhealthy eating habits differed greatly between nurses and physicians, where nurses declared this to a higher degree. Health professionals with less than 10 years in their profession had increased their work with hazardous use of alcohol. The overall extent to which both groups worked with the different lifestyles was reported as follows: tobacco use (78%); insufficient physical activity (77%); unhealthy eating habits (68%); and hazardous use of alcohol (65%). Furthermore, 73 % of the health professionals would like to work with patients’ lifestyles to a higher extent than today. However, compared to the first measurement, the physicians reported this to a lower degree and nurses were more favourable than physicians were. Lastly, 55 % considered that promotion of healthy lifestyle habits was a substantial part of their duties and also here, nurses reported this to a higher degree than physicians did.

#### Organizational support structures

No differences over time, by profession or by years in profession were found (Table 4).

**Table 4:** Organizational support structures for clinical work with patients lifestyles, percent of health professionals who agree to a high extent

Statements	At my PHCC, management is positive toward our work with promotion of healthy lifestyles					At my PHCC, we collaborate with other stakeholders such as municipalities and community associations about our patients lifestyles				
Questionnaire	Q I		Q II		Q I → Q II	Q I		Q II		Q I → Q II
Characteristics	%	P	%	P	P	%	P	%	P	P
Profession		0.256		0.295			0.066		0.484	
Nurses	75		74		0.740	26		21		0.372
Physicians	69		64		0.638	15		15		0.988
Years in profession		0.552		0.254			0.422		0.298	
0–10	76		63		0.162	20		25		0.539
> 10	72		73		0.815	25		17		0.167
Total	73		70		0.560	23		19		0.426
Statements	At my PHCC, there are incentives to promote healthy lifestyles among our patients					At my PHCC, there are local guidelines/care programs on how we should promote healthy lifestyles among our patients				
Questionnaire	Q I		Q II		Q I → Q II	Q I		Q II		Q I → Q II
Characteristics	%	P	%	P	P	%	P	%	P	P
Profession		0.924		0.740			0.153		0.567	
Nurses	65		72		0.261	58		57		0.870
Physicians	65		69		0.673	48		51		0.736
Years in profession		0.036		0.412			0.596		0.276	
0–10	56		66		0.353	52		47		0.593
> 10	70		73		0.601	56		58		0.762
Total	65		71		0.248	55		55		0.972

*PHCC* Primary health care centre

*PHC* Primary health care

*QI* Questionnaire I (May, 2011)

*QII* Questionnaire II (May, 2013)

Management at the PHCC was confirmed to be positive to work with promotion of healthy lifestyles, and 71% stated that there are incentives to promote such work. The respondents referred to a low level of collaboration around patients’ lifestyles with other stakeholders, consistent with the previous measurement. The health professionals reported on existing and available local guidelines/care programs on how to promote healthy lifestyles.

## Discussion

### Implementation

The degree of implementation of the Swedish CPGs on life style interventions is so far discrete, only 18% of the physicians and 36% of the nurses reported that the CPGs had been implemented to a high degree.

#### Differences between physicians and nurses in uptake of CPGs

In general, there is an obvious difference in physicians’ and nurses’ attitudes to and use of the CPGs. While the nurses find the CPGs as a support in their work and a majority always use them, less than one fifth of the physicians use them regularly. In fact, for the use of every single support tool there is a significant difference between physicians and nurses. It is known that physicians’ uptake and compliance rate of any clinical guideline vary [27–29] and that compliance rate with guidelines is influenced by the target area of the guidelines [30]. Our data were collected 18 months after the release of the guidelines and the implementation cannot be considered as completed. There is still the possibility that physicians’ uptake will improve, but the low level of use and appreciation of the guidelines as support are concerns that should be considered when planning for further implementation activities.

#### Perceived implementation obstacles

The qualitative results point out some perceived implementation obstacles worthy to be taken into account: the physicians report about a general tiredness of guidelines in primary care, structural factors like medical records not being adapted to the CPGs, and a heavy workload with a shortage of time resulting in lifestyle counselling being considered as too time consuming. The lack of time is a common obstacle to implementation referred to in several studies [6, 10, 13].

#### Shortage of workforce

Over and above time constraints, the respondents referred to the lack of continuity due to shortage of physicians and/or use of temporarily physicians as an important obstacle to the implementation. It is known that smooth implementation is more likely in stable teams with low turnover [2] and therefore it is possible that the reported shortage of the health professionals and temporary workforce could hamper the implementation. Furthermore, the respondents refer to the size of the PHCC and the numerous workforces as being an obstacle to implementation. Evidence shows that structural characteristics of the organization like size positively influence implementation. For example, a large organization can access additional resources for development and assumes functional differentiation with a higher ratio of managers more easily than a small-sized organization [3]. Primary care centers are often organised as independent rather small administrative units with a single manager responsible for the quality of the care provided, economy, and staff. This might affect implementation.

#### Lack of priority

In addition, the respondents in this study refer to the field of the guidelines as not being prioritised, and that work with patients’ lifestyles is being done “on top of the rest”. This data is in accordance with previous studies about how difficult it is to prioritize prevention and health promotion in health care and that traditional “medical care” is prioritised on the cost of health promotion, which continues to be optional and not so well integrated [6, 7, 13, 14].

### Follow-up of work with lifestyle interventions in primary healthcare

Our findings show few changes over time. However, there are important changes concerning the extent of work with different lifestyles. Compared to the first measurement, there is a significant and positive change in the usage of counselling to aid changes in patients’ unhealthy lifestyles concerning tobacco use and hazardous use of alcohol. Still, alcohol remains to be the least addressed lifestyle of the four, which is consistent with the first study and will need further investigation. Nurses have significantly increased the extent to which they work with each lifestyle compared to the first study. Despite this, there are no significant differences in extent of work with the different lifestyles between physicians and nurses in the second measurement, except for unhealthy eating habits. Nurses however, in general considered the promotion of patient lifestyle as a substantial part of their work to a higher degree than physicians did (no difference from the first study). Is the increase in nurses’ extent of work a result of the ongoing implementation of the CPGs on lifestyle interventions? And if so, how come the physicians’ level of involvement has not increased?

In our study, both physicians and nurses showed a positive attitude to work with patients’ lifestyles. However, physicians referred significantly lower than in the first study that they would like to work to a greater extent with this issue despite that they did not increased their extent of work with patients’ lifestyles between the two measurements periods. Attitude is important for an effective implementation [3, 4], and the decline in the physicians’ interest to further development needs consideration.

Furthermore, the health professionals’ knowledge level is a key factor to a successful implementation [3], and the lack of it could hinder the development. In this study, the level of knowledge continues to be reported as high across the health professional groups. Likewise, the knowledge of methods to aid patients’ lifestyle change concerning alcohol remains to be the lowest of the four different lifestyles. The identified gap between physicians and nurses concerning knowledge of methods to aid patients’ to change their unhealthy eating habits in the first study was confirmed. Knowledge of counselling, an important skill needed to be able to follow the guideline recommendations, was not measured in the first study. In the second survey, half of the physicians and 37% of the nurses reported to have a thorough knowledge in counselling. Literature emphasizes the importance of skill development and ease of access to information about the intervention for implementation outcomes [2, 3]. Thus, even if the knowledge level in general can be considered as high within the organisation, more targeted education efforts seems to be needed concerning e.g. methods for reducing the hazardous use of alcohol, counselling skills and physicians’ knowledge of unhealthy eating habits.

Moreover, engaged leaders are important to implementation [2] and the health professionals continue to report about managers that are positive to the work with patients’ lifestyles and that there are incentives to carry out this work. Lastly, the PHCCs’ collaboration with other stakeholders (e.g. sport clubs, patient associations, municipalities etc.) in the community remains low, only 15% of the health professionals reported having such relationships. Previous studies have described how the promotion of lifestyles can suffer from a lack of referral options to other professionals [11] and that it is desirable to develop these kind of relations [25].

### Strengths and limitations

This study is, as far as we know, the first longitudinal study that investigate the uptake of national CPGs on lifestyle interventions and the change over time in work with lifestyle counselling in clinical practice. It contributes with knowledge of health professional groups’ attitudes, level of knowledge, extent of work and organisational support for patients’ lifestyle change.

The generalisability of the results is subject to certain limitations. For instance, the internal validity could partly be biased due to the response-rate and the nature of self-reported data. The overall response rate in the present study was 50% and these individuals were part of the 49% who answered the first questionnaire. During 1996 to 2005, questionnaires to health professionals in general were answered by 56% [31] and participation in epidemiologic studies, like cohorts, are declining [32]. The response-rate in the present study is therefore in line with other similar studies. There may be response bias because those who responded may have agreed more with the guidelines, and/or individuals interested in health orientation might have answered the questionnaires to a higher degree. Non-response bias could not be assessed because there were not any details on the non-responders. Furthermore, it is a limitation that a one of the healthcare organisations was lost between the first questionnaire and the follow up in the second questionnaire. Moreover, the results need to be interpreted with caution due to the nature of self-reported data since it can be biased by the respondents’ exaggerations, feelings, social desirability etc. [33, 34]. Furthermore, external validity is limited since the study investigated one of 21 healthcare organisations in Sweden, and it is not possible to generalize the results to other settings. The chosen organisation was also known to previously have focused on health orientation, which might have contributed to an overestimation of the positive findings.

The two-year period might have been too short to draw any major conclusions about the uptake of the guideline implementation – but can be seen as a first assessment of the same as well as indicating potential obstacles, which can be useful for further planning of the implementation of the CPGs.

## Conclusions

This study has shown that even if the uptake and usage of the CPGs on lifestyle interventions so far is low, the participants reported more frequent counselling on patients’ lifestyle changes concerning use of tobacco and hazardous use of alcohol 2 years after the introduction of the CPGs. However, these findings should be evaluated acknowledging the possibility of selection bias in favour of health promotion and lifestyle guidance, and the loss of one study site in the follow up. Furthermore, this study indicates important differences in physicians and nurses’ attitudes to and use of the guidelines, where the nurses reported working to a higher extent with all four lifestyles compared to the first study. There is an uncertainty concerning physicians’ involvement in lifestyle counselling and attitudes concerning further development of the field. These findings suggest a need for further investigations on the implementation and uptake of the CPGs, and especially the physicians’ use of the CPGs.

## Additional file

Additional file 1: Questionnaire. (DOCX 19 kb)

## Abbreviations

CPGs: Clinical Practice Guidelines
NBHW: National Board of Health and Welfare
PHC: Primary Health Care
PHCC: Primary Health Care Centre
