# Supplementary material for: Implementation of clinical practice guidelines on lifestyle interventions in Swedish primary healthcare – a two-year follow up
Source: BMC Health Serv Res. 2018 Apr 2;18:227. doi: 10.1186/s12913-018-3023-z (PMC5880081; doi:10.1186/s12913-018-3023-z)
Supplement: Supplementary file 1 — Questionnaire. (DOCX 19 kb) [file 12913_2018_3023_MOESM1_ESM.docx]

Additional file 1: Questionnaire I^[[1]](#footnote-1)^

| 1.Sex |
| --- |
| 2.My present job title is: |
| 3.With my present title I’ve been working: |
| 4. At my PHCC, there is a strategy about receiving and implementing new knowledge |
| 5. At my PHCC, priority is given to competence development and continuous education |
| 6. At my PHCC, there are well functioning information and communication channels |
| 7. At my PHCC, we have a great support from management in work with change and development |
| 8. At my PHCC, there is an openness and possibility to try new ways of working |
| 9. At my PHCC, I have good opportunities to influence the organisations development |
| 10. At my PHCC, I have good opportunities to influence my work duties and the way I’m working. |
| 11. At my PHCC, we have a thorough knowledge of how we can promote healthy lifestyle habits among our patients |
| 12. At my PHCC, we consider it important to promote healthy lifestyles for our patients |
| 13. At my PHCC, there are incentives to promote healthy lifestyles among our patients |
| 14. At my PHCC, there are local guidelines/care programs on how we should promote healthy lifestyles among our patients |
| 15. At my PHCC, we work extensively with the promotion of healthy lifestyles among our patients |
| 16. At my PHCC, there are many factors that facilitate the promotion of healthy lifestyles among our patients |
| 17. At my PHCC, there are many factors that hinder the promotion of healthy lifestyles among our patients |
| 18. At my PHCC, management is positive toward our work with promotion of healthy lifestyles |
| 19. At my PHCC, we collaborate with other stakeholders such as municipalities and community associations about our patients’ lifestyles |
| 20. At my PHCC, the recommendations in different national guidelines, are important as support tools concerning the respective disease/condition |
| 21. At my PHCC, there is a strategy/modell to implement national guidelines |
| 22a. At my PHCC, the responsibility to implement new national guidelines is: a) health organization’s management |
| 22b. At my PHCC, the responsibility to implement new national guidelines is: b) PHCC management |
| 22c. At my PHCC, the responsibility to implement new national guidelines is: c) all employees |
| 23. At my PHCC, there are many factors that facilitate the implementation of new national guidelines |
| 24. At my PHCC, there are many factors that hinder the implementation of new national guidelines |
| 25. At my PHCC, we have particular resources for the implementation of national guidelines |
| 26. At my PHCC, we have support from our manager to work with national guidelines |
| 27. At my PHCC, national guidelines increase the quality of care |
| 28. At my PHCC, we update our local care programs according to recommendations in national guidelines |
| 29. At my PHCC, the use of national guidelines is controlled in our collective regular follow-ups |
| 30. I don’t think CPGs on methods for disease prevention are needed |
| 31. I always make note about my patients lifestyle habits in the medical record |
| 32 I would like to work to a greater extent with promotion of healthy lifestyles among my patients |
| 33a I have a thorough knowledge of disease prevention methods concerning tobacco use |
| 33b I have a thorough knowledge of disease prevention methods concerning hazardous use of alcohol |
| 33c I have a thorough knowledge of disease prevention methods concerning unhealthy eating habits |
| 33d I have a thorough knowledge of disease prevention methods concerning insufficient physical activity |
| 34. I consider the promotion of healthy lifestyle habits to patients in health care as being cost effective |
| 35a I work to a great extent with promotion of healthy lifestyle habits concerning tobacco use |
| 35b I work to a great extent with promotion of healthy lifestyle habits concerning hazardous use of alcohol |
| 35c I work to a great extent with promotion of healthy lifestyle habits concerning unhealthy eating habits |
| 35d I work to a great extent with promotion of healthy lifestyle habits concerning insufficient physical activity |
| 36. I consider duties concerning lifestyle habits as compatible with PHCs aim and objectives |
| 37. I perceive a need of CPGs on methods for disease prevention in my work |
| 38. I consider the implementation of CPGs on methods for disease prevention as challenging for our present way of working at the PHCC |
| 39. I’m very worried about the change in my work that the implementation of CPGs on methods for disease prevention will bring about |
| 40. I think the implementation of CPGs on methods for disease prevention will hamper my work |
| 41. For me it is easy to develop and try new ways of working and new routines |
| 42. I perceive a need to develop PHCs’ work to promote healthy lifestyle habits in our patients |
| 43. I think my PHCC will benefit from the implementation of CPGs on methods for disease prevention |
| 44. I think my direct heads of department will hesitate to support the implementation of CPGs on methods for disease prevention |
| 45. The promotion of healthy lifestyle habits among my patients is a substantial part of my duties |
| 46. The objectives of the PHCC are clear |
| 47. To me it’s important to receive support from my direct head of department in trying to develop the work |
| 48. In general, I am positive to national guidelines in health care |
| 49 Within my work in PHC I’ve been working with the following guidelines: |
| 50 Other: |

PHC - Primary Health Care

PHCC - Primary Health Care Centre

CPGs - Clinical Practice Guidelines

1. This questionnaire was originally presented to participants in Swedish, but was translated to English for this article. [↑](#footnote-ref-1)
